# Bases for Artificial Teeth

**Published:** 1870-04

**Authors:** 


### BASES FOR ARTIFICIAL TEETH.

Editors Register:—Your reference to "Bases for Artificial Teeth," found on page forty-eight (48) of last Register, is full of good things, and allow me to say, that during the past year I have done but very little plate work where I used any base but gold, porcelain, aluminum or silver, and it is with pride and pleasure that I know that I have made better operations, satisfied my patients better, and last, but not least, I have made money by this policy. I have the happy satisfaction, also, of knowing that my patients have a confidence in my judgment to a much greater extent than I had before supposed.

We (many of us) have, in by gone years, been too timid about standing up for our own opinions and convictions, and to what was best to be used to retain the natural teeth, as well as that which was the most durable and comfortable to be worn in the mouth, as a retainer of artificial teeth. But let us now look to it, that we, from this time forward, consult our own knowledge and convictions of what is right toward our patients and ourselves. Then we will inspire in them that confidence, which will elevate our profession in their judgement, and so make us better operators, better men, and infinitely more happy.

As to low priced dentures, I will only say, that the best that I have ever found is aluminum plate Starr's solder, *this I have used to my entire satisfaction*, and only hope that Watt and Williams, of Xenia, O., have something that is *better*, but I can scarcely hope for this, as Starr's mode of operating in mounting teeth is easy to any good Dentist, and recently I had the positive assertion of that most eminent "Dental chemist," Dr. Geo. Watt, of Cincinnati, that it was very much the best low priced denture ever worn.

Zanesville, O. W. M. H.

